# C-Myc regulation by costimulatory signals modulates the generation of CD8^+^ memory T cells during viral infection

**DOI:** 10.1098/rsob.150208

**Published:** 2016-01-20

**Authors:** Mohammad Haque, Jianyong Song, Kristin Fino, Youfei Wang, Praneet Sandhu, Xinmeng Song, Christopher Norbury, Bing Ni, Deyu Fang, Shahram Salek-Ardakani, Jianxun Song

**Affiliations:** 1 Department of Microbiology and Immunology, The Pennsylvania State University College of Medicine, Hershey, PA, USA; 2 Institutes of Irradiation/Immunology, The Third Military Medical University, Chongqing, People's Republic of China; 3 Department of Pathology, Northwestern University Feinberg School of Medicine, Chicago, IL, USA; 4 Department of Pathology, Immunology and Laboratory Medicine, University of Florida, Gainesville, FL, USA

**Keywords:** c-Myc, costimulation, CD8^+^ T cells, memory, viral infection

## Abstract

The signalling mechanisms of costimulation in the development of memory T cells remain to be clarified. Here, we show that the transcription factor c-Myc in CD8^+^ T cells is controlled by costimulatory molecules, which modulates the development of memory CD8^+^ T cells. C-Myc expression was dramatically reduced in *Cd28*^−/−^ or *Ox40^−/−^* memory CD8^+^ T cells, and c-Myc over-expression substantially reversed the defects in the development of T-cell memory following viral infection. C-Myc regulated the expression of survivin, an inhibitor of apoptosis, which promoted the generation of virus-specific memory CD8^+^ T cells. Moreover, over-expression of survivin with bcl-xL, a downstream molecule of NF-*κ*B and intracellular target of costimulation that controls survival, in *Cd28*^−/−^ or *Ox40^−/−^* CD8^+^ T cells, reversed the defects in the generation of memory T cells in response to viral infection. These results identify c-Myc as a key controller of memory CD8^+^ T cells from costimulatory signals.

## 1. Introduction

T-cell memory is a fundamental characteristic of adaptive immunity. A typical T-cell response to a viral infection undergoes an expansion phase culminating in the generation of effector T cells, a contraction/death phase when over 90% of the effector T cells undergo apoptosis, and a memory phase that is maintained long-term in the absence of viral antigen (Ag). After a primary immune reaction following the initial viral infection, a small pool of virus-specific memory T cells (approx. 1–5% of peak expansion) is retained for protection against re-infection. As a result, T-cell memory is an integral part of a protective immunity against viral infections, and understanding their development is needed for designing optimal vaccines [1,2].

A number of key components required for the generation of memory CD8^+^ T cells are known. During the expansion phase, Ag stimulation affects the burst size, such that a larger effector T-cell population induced can form a larger memory T-cell pool. During the expansion and/or contraction phases, costimulatory signalling from CD28 or TNFR family members (e.g. CD28, CD27, OX40, 4-1BB) can reduce death of effector cells by providing pro-survival signals [3,4]. When entering the memory phase, memory T progenitors need IL-7 and IL-15 to maintain the quality and quantity of memory T cells [5,6]. However, additional factors regulating cell expansion/death, and homeostatic proliferation during the development of memory T cells remain to be identified.

The mechanisms that control the development of memory T cells have been studied, and various transcription factors that regulate the development of effector and memory CD8^+^ T cells have been identified. It has been established that transcription factors, such as Eomesodermin (EOMES) [7], the forkhead O transcription factor 1 (Foxo1) [8], the inhibitor of DNA binding 3 (ID3) [9], T-cell factor 1 (Tcf1) [10], and the signal transducer and activator of transcription-3 (Stat3) [11], prevent terminal differentiation and/or help maintain the fundamental properties of memory T cells. These fundamental properties include long-term survival, a high proliferative potential, the developmental plasticity and the ability to self-renew [12]. These transcription factors may have an overall role during the development of memory CD8^+^ T cells; however, the transcriptional regulation is likely to vary during the various phases of differentiation and needs to be further elucidated.

We previously identified survivin, an inhibitor of apoptosis family protein, and aurora B as intracellular targets of OX40 and/or CD28. In T cells, aurora B complexes with survivin [13,14]. We recently showed that the transgenic expression of survivin promotes T-cell proliferation and persistence [15,16], resulting in an enhanced development of memory CD8^+^ T cells. These observations raised the possibility that survivin might be regulated by the transcription factors from costimulatory signals during the expansion and/or contraction phases of memory T cells, and that this regulation might impact the quality and quantity of memory CD8^+^ T cells.

Here, we demonstrated that the defective development of memory CD8^+^ T cells in *Cd28*^−/−^ or *Ox40*^−/−^ mice was caused by the reduced expression of two transcriptional factors c-Myc and Nfkb1 during viral infection. Over-expression of c-Myc or IKKβ that compensates for the deficiency of Nfkb1 in canonical NF-*κ*B activity in these CD8^+^ T cells could substantially reverse the defect in the development of memory CD8^+^ T cells. In addition, c-Myc controlled survivin expression and regulated T-cell memory. Of note, over-expression of survivin, the downstream target of c-Myc, with bcl-xL, a downstream survival molecule of NF-κB [17,18] in *Cd28*^−/−^ or *Ox40*^−/−^ T cells restored defective responses of memory T cells. Our results define c-Myc, together with Nfkb1, as two key transcriptional regulators of memory CD8^+^ T cells from costimulatory signals.

## 2. Results

### 2.1. A critical role of costimulatory signals in the generation of memory CD8^+^ T cells

**Figure 1.**
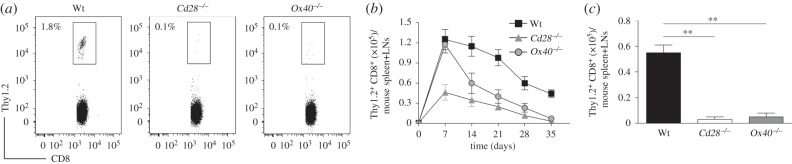
*Cd28*^−/−^ or *Ox40^−/−^* T cells are defective in the generation of memory CD8^+^ T cells. 1 × 10^3^ naive Thy1.2 CD8^+^ TCRVβ5^+^ T cells from OT-I, OT-I/*Cd28*^−/−^ or OT-I/*Ox40^−/−^* mice were adoptively transferred into Thy1.1 congenic mice infected *i.p.* with VV-OVA (2 × 10^6^ pfu mouse^−1^). After various days, the transferred T cells from the spleen and LNs were analysed. Five mice were used for each time point. (*a*) The frequencies of Thy1.2^+^ T cells at day 35 post-infection of VV-OVA, gating on CD8^+^ cells. Data are representative of two independent experiments (*p* < 0.001 between Wt and *Cd28*^−/−^ or *Ox40^−/−^*; one-way ANOVA). (*b*) Numbers of CD8^+^ Thy1.2^+^ T cells at indicated day post-infection of VV-OVA. Data are represented as the mean ± s.e.m. (*p* < 0.001 on days 7, 14, 21, 28 and 35, and *p* < 0.05 on day 21 between Wt and *Cd28*^−/−^; *p* < 0.05 on day 7 and *p* < 0.001 on days 14, 21, 28 and 35 between Wt and *Ox40*^−/−^; *p* < 0.001 on days 7 and 14, and *p* < 0.05 on day 21 between *Cd28*^−/−^ and *Ox40*^−/−^; two-way ANOVA). (*c*) Numbers of CD8^+^ Thy1.2^+^ T cells at day 35 post-infection of VV-OVA. Data are represented as the mean ± s.e.m. from two independent experiments (**p < 0.01; one-way ANOVA).

To confirm the role of costimulatory signals in the generation of Ag-specific memory CD8^+^ T cells, we used OT-I TCR-transgenic mice and vaccinia virus expressing ovalbumin (VV-OVA). Naive Thy1.2 CD8^+^ TCRVβ5^+^ T cells from OT-I (wild-type; Wt), OT-I/*Cd28*^−/−^ (*Cd28*^−/−^) and OT-I/*Ox40^−/−^* (*Ox40*^−/−^) mice were adoptively transferred into Thy1.1 congenic mice which were then infected *i.p.* with VV-OVA. At day 35, post-infection of VV-OVA, virus-specific memory CD8^+^ T cells from the spleens and LNs of mice were determined by gating on CD8^+^ Thy1.2^+^ populations. Compared with the recipients receiving the transferred T cells from Wt mice, the frequencies of virus-specific memory CD8^+^ T cells were significantly reduced in the recipients receiving the T cells from *Cd28*^−/−^ or *Ox40*^−/−^ mice (1.8% versus 0.1%; figure 1*a*). After 7 or 35 days, transferred CD8^+^ Thy1.2^+^ T cells from the spleens and LNs of the recipients were analysed. The frequency of OVA-specific CD8^+^ T cells in the recipients receiving the T cells from *Cd28*^−/−^ or *Ox40*^−/−^ mice was dramatically lower from days 7 to 35 post-infection with VV-OVA in comparison to the recipients receiving the transferred T cells from OT-I mice. However, the number of OVA-specific CD8^+^ T cells in mice receiving *Ox40*^−/−^ T cells at day 7 was similar in mice receiving Wt mice, but dramatically reduced at days 14 to 35 (figure 1*b*,*c*). Collectively, these results demonstrated that costimulatory signals play a critical role in the generation of memory CD8^+^ T cells during the primary viral infection.

### 2.2. Transcriptional regulation of costimulatory signals in the generation of memory CD8^+^ T cells

**Figure 2.**
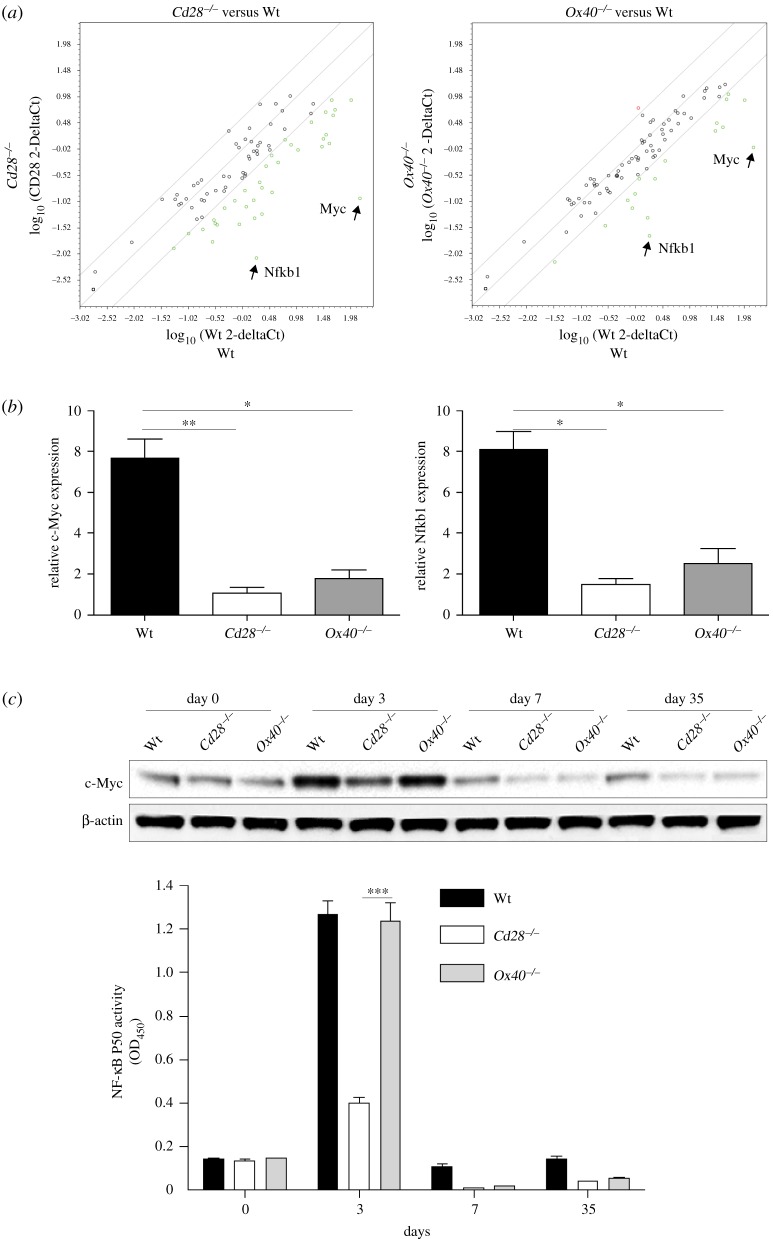
Transcription of c-Myc and Nfkb1 is reduced in *Cd28*^−/−^ or *Ox40^−/−^* memory CD8^+^ T cells. Naive Thy1.2 CD8^+^ TCRVβ5^+^ T cells from OT-I, OT-I/*Cd28*^−/−^ or OT-I/*Ox40^−/−^* mice were adoptively transferred into Thy1.1 congenic mice infected *i.p.* with VV-OVA. At day 35, approximately 1 × 10^6^ Thy 1.2^+^ memory T cells from the spleen and LNs were sorted. (*a*) The scatter plots of PCR array. Total RNA (1 µg) was isolated for the RT^2^ Profiler PCR Array. Pairwise comparison of Wt and *Cd28*^−/−^ or *Ox40^−/−^* by scatter plot analysis. Spots associated with individual transcription factor genes were collected and converted into a log_10_ scale. The central line indicates unchanged gene expression. The dots are allocated to positions that are above or below than the +3 fold or −3 fold line when the differences are greater than threefolds. Data are representative of three independent experiments. (*b*) Relative expression of c-Myc and Nfkb1 by RT-PCR. Data are represented as the mean ± s.e.m. from three independent experiments (*p < 0.05, **p < 0.01. One-way ANOVA). (*c*) Protein expression of c-Myc and canonical NF-κB activity detected by immunoblot or p50 ELISA. Similar data were obtained in three experiments (***p < 0.001; two-way ANOVA). In Western blots, β-actin was used as internal control. For Western blots belonging to the same experiment, bands pertaining to different proteins were cropped from the same blot.

To understand the regulation of costimulatory signals in the generation of memory CD8^+^ T cells, we performed PCR Arrays and analysed the expression of a focused panel of transcription factor genes. Naive Thy1.2 CD8^+^ TCRVβ5^+^ T cells from Wt *Cd28*^−/−^ or *Ox40^−/−^* mice were adoptively transferred into Thy1.1 congenic mice which were then infected *i.p.* with VV-OVA. At day 35 post-infection of VV-OVA, Thy 1.2^+^ CD8^+^ donor memory T cells from the spleen and LNs were sorted. Gene expression of transcriptional factors was analysed using the RT^2^ Profiler PCR Array. Compared with OVA-specific memory CD8^+^ T cells from Wt donors, memory T cells from *Cd28*^−/−^ or *Ox40*^−/−^ donors had a substantially lower expression of c-Myc and Nfkb1 from the screened 84 genes within the panel of transcription factors (figure 2*a*). C-Myc is activated by various signals such as Wnt via the MAPK/ERK pathway [19], and c-Myc activation modified the expression of target genes that results in a number of biological effects, including cell proliferation, cell growth, apoptosis, differentiation, metabolic reprogramming and stem cell self-renewal.

Nfkb1 encodes 105 kD protein, which can undergo co-translational processing by the 26S proteasome to produce a 50 kD protein. The 105 kD protein is a Rel protein-specific transcription inhibitor and 50 kD protein is a DNA-binding subunit of NF-κB, which plays a key role in regulating the immune response to infection. To confirm the results of the RT^2^ Profiler PCR Array, RT-PCR was performed on OVA-specific memory CD8^+^ T cells from Wt *Cd28*^−/−^ or *Ox40*^−/−^ donors. Compared with the OVA-specific memory CD8^+^ T cells from Wt donors, we observed that memory T cells from *Cd28*^−/−^ or *Ox40*^−/−^ donors had significantly reduced upregulation in the mRNA expression of c-Myc and Nfkb1 at day 35 post-infection of VV-OVA (figure 2*b*). These results suggest transcriptional regulation of signals from CD28 or OX40 in the generation of memory CD8+ T cells during VV-OVA infection.

We also analysed the protein expression of c-Myc by immunoblots and canonical NF-κB activity by a p50 ELISA in memory CD8^+^ T cells. Similar to mRNA expression, the overall protein expression of c-Myc and canonical NF-κB activity was higher in memory CD8^+^ T cells from Wt OT-I donors than from *Cd28*^−/−^ or *Ox40*^−/−^ donors (figure 2*c*). Given the importance of c-Myc and Nfkb1 in the biological effects, the defects in the expression of c-Myc and Nfkb1 may affect the quantity of virus-specific memory CD8^+^ T cells in the absence of CD28 and OX40. This is in line with data from mice lacking CD28 or OX40 signals, which show that such deficiency can reduce the quantity of virus-specific memory CD8^+^ T cells [20,21]. Collectively, these results indicate that the c-Myc and Nfkb1 transcriptional regulation of costimulatory signals is likely to modulate the generation of virus-specific memory CD8^+^ T cells to viral infection.

### 2.3. C-Myc promotes the generation of memory CD8^+^ T cells during an interrogation of primary response

**Figure 3.**
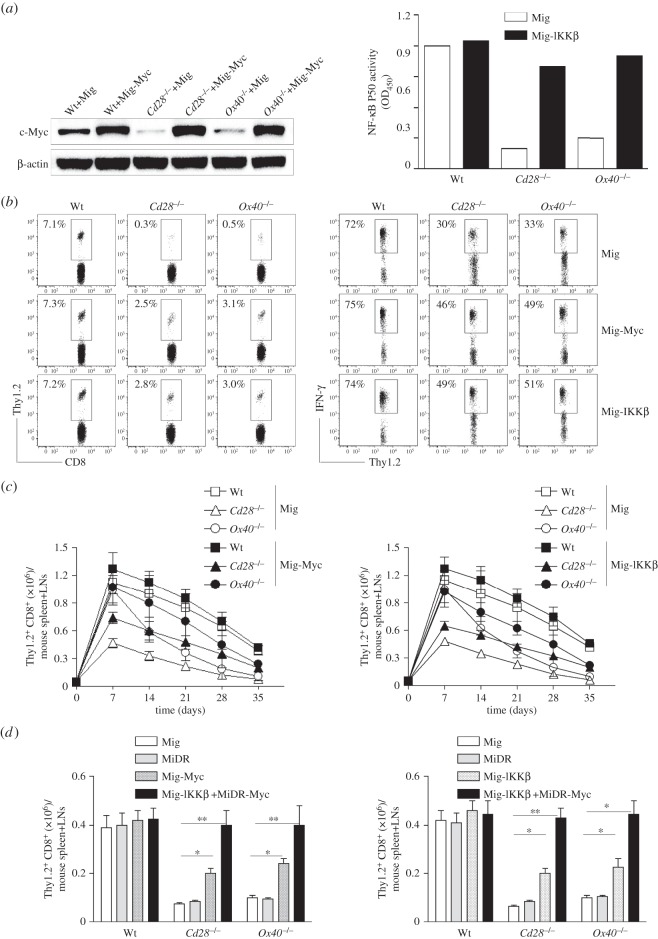
Over-expression of c-Myc or IKKβ substantially reverses the defective generation of *Cd28*^−/−^ or *Ox40^−/−^* memory CD8^+^ T cells during an interrogation of primary response. Naive Thy1.2 CD8^+^ TCRVβ5^+^ T cells from OT-I, OT-I/*Cd28*^−/−^ or OT-I/*Ox40^−/−^* mice were stimulated with peptide and APCs. On day 2/3, T cells were transduced with retroviral vectors expressing GFP alone (Mig), GFP with c-Myc (Mig-Myc), GFP with CA-IKKβ (Mig-IKKβ) or DsRed with c-Myc (MiDR-Myc) plus Mig-IKKβ. On day 5 of primary culture, 5 × 10^4^ GFP^+^ or GFP^+^ DsRed^+^ CD8 cells were sorted and adoptively transferred into Thy1.1 congenic mice that were infected *i.p.* with VV-OVA on the following day. After various days, the transferred T cells from the spleen and LNs were analysed. Five mice were used for each time point. (*a*) C-Myc or ΙKKβ transduction. On day 5 of primary culture, GFP^+^ T cells were sorted and analysed for c-Myc and β-actin or canonical NF-κB activity by immunoblot or p50 ELISA. Similar data were obtained in three experiments (*p* < 0.001 between Mig and Mig-IKKβ in *Cd28*^−/−^ or *Ox40^−/−^*; two-way ANOVA). In Western blots, β-actin was used as internal control. For Western blots belonging to the same experiment, bands pertaining to different proteins were cropped from the same blot. (*b*) The frequencies of Thy1.2^+^ T cells at day 35 post-infection of VV-OVA, gating on CD8^+^ cells (left). At day 35 post-infection of VV-OVA, splenocytes were stimulated with OVA peptide for intracellular IFN-γ staining, gating on Thy1.2^+^ cells (right). Data are representative of three independent experiments (*p* < 0.001 between Wt and *Cd28*^−/−^ or *Ox40^−/−^* with Mig; *p* < 0.01 between Wt and *Cd28*^−/−^ or *Ox40^−/−^* with Mig-Myc or Mig-IKKβ both left and right panels; *p* < 0.001 between Mig and Mig-Myc or Mig-IKKβ in *Cd28*^−/−^ or *Ox40^−/−^*; *p* < 0.05 between Mig and Mig-Myc or Mig-IKKβ in *Cd28*^−/−^ or *Ox40^−/−^* both left and right panels; two-way ANOVA). (*c*) Numbers of CD8^+^ Thy1.2^+^ T cells at indicated day post-infection of VV-OVA. Data are represented as the mean ± s.e.m. (*p* < 0.01 between Mig and Mig-Myc in *Ox40^−/−^* on days 14 and 21; *p* < 0.05 between Mig and Mig-IKKβ in *Ox40^−/−^* on days 14, 21 and 28; two-way ANOVA). (*d*) Numbers of CD8^+^ Thy1.2^+^ T cells at day 35 post-infection of VV-OVA. Data are represented as the mean ± s.e.m. from three independent experiments (*p < 0.05, **p < 0.01; two-way ANOVA).

To determine if a lack of c-Myc or Nfkb1 was responsible for the observed defective generation of *Cd28*^−/−^ or *Ox40*^−/−^ memory CD8^+^ T cells, we retrovirally transduced T cells with c-Myc or active CA-IKKβ that increases NF-κB activation and nuclear translocation. Over-expression of active CA-IKKβ can augment p105 protein to be processed to active p50 form and enhance canonical NF-κB activity [17,22], which is likely to reverse the defect of canonical NF-κB activity by the reduced expression of Nfkb1. Naive Thy1.2 CD8^+^ TCRVβ5^+^ T cells from Wt *Cd28*^−/−^ or OT-I/*Ox40^−/−^* mice were stimulated with OVA peptide and APCs. On day 2/3, T cells were transduced with retroviral vectors expressing GFP alone (Mig), GFP with c-Myc (Mig-Myc) or GFP with CA-IKKβ (Mig-IKKβ). On day 5 of primary culture, GFP^+^ CD8 cells were sorted, and over-expression of c-Myc or reversion of canonical NF-κB activity was confirmed by immunoblots or a p50 ELISA (figure 3*a*). GFP^+^ CD8 cells were adoptively transferred into Thy1.1 congenic mice that were infected *i.p.* with VV-OVA on the following day. At day 35 post-infection of VV-OVA, virus-specific memory Thy1.2^+^ T cells from the spleen and LNs of mice were determined, gating on CD8^+^ cells. The decrease in numbers of virus-specific memory cells from *Cd28*^−/−^ or *Ox40*^−/−^ mice was significantly reversed with over-expression of c-Myc or CA-IKKβ (figure 3*b*).

To evaluate the function of the memory T cells from *Cd28*^−/−^ or *Ox40*^−/−^ CD8^+^ T cells expressing c-Myc or CA-IKKβ, at day 35 post-infection of VV-OVA, splenocytes were stimulated with OVA peptide for intracellular IFN-γ staining, gating on Thy1.2^+^ cells. This functional deficiency of virus-specific memory cells from *Cd28*^−/−^ or *Ox40*^−/−^ CD8^+^ T cells were partially rescued when over-expressing c-Myc or CA-IKKβ (figure 3*b*).

To show that the expression of c-Myc or CA-IKKβ alone could reverse defective development of memory CD8^+^ T cells in *Cd28*^−/−^ or *Ox40*^−/−^ T cells, we assessed the frequencies of transferred CD8^+^ T cells during viral infection. The low frequencies of virus-specific T cells from *Cd28*^−/−^ or *Ox40*^−/−^ mice on days 7 to 35 were significantly improved when over-expressing c-Myc or CA-IKKβ (figure 3*c*), and completely reversed when over-expressing both c-Myc and CA-IKK (figure 3*d*).

Taken together, these observations support the hypothesis that the transcriptional regulation of c-Myc and Nfkb1 from costimulatory signals strongly modulates the expansion and development of virus-specific memory CD8^+^ T cells during an interrogation of primary response.

### 2.4. C-Myc promotes the generation of memory CD8^+^ T cells during the primary immune response

During the primary viral infection, *Cd28*^−/−^ or *Ox40*^−/−^ mice developed a small number of memory CD8^+^ T cells compared to Wt mice (figure 1). In addition, the progenitor memory and memory T cells in these *Cd28*^−/−^ or *Ox40*^−/−^ mice had a reduced expression of c-Myc (figure 2). We decided to test whether over-expression of c-Myc in naive T cells could compensate for the defective generation of memory CD8^+^ T cells during a primary immune response.

**Figure 4.**
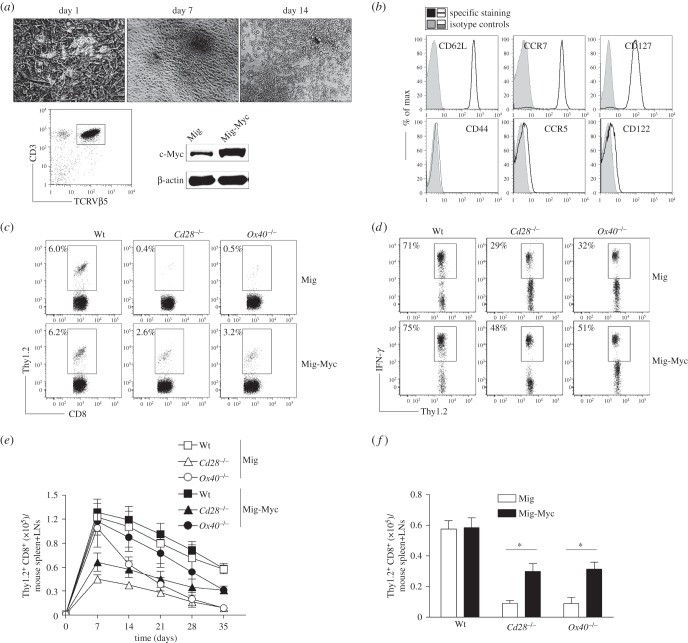
Over-expression of c-Myc substantially reverses the defective memory generation of *Cd28*^−/−^or *Ox40^−/−^* CD8^+^ T cells during the primary immune response. CD117^+^ HSCs from the bone marrows of OT-I, OT-I/*Cd28*^−/−^ or OT-I/*Ox40^−/−^* mice were cultured on SNL feeder cells and transduced with retroviral vectors expressing GFP alone, or GFP with c-Myc. GFP^+^ HSCs were sorted and cultured on OP9-DL1/DL4 cells expressing Notch ligands DL1 and DL4 in the presence IL-7 and Flt3 L. After 14 days, 1 × 10^3^ CD3^+^ TCRβ5^+^ progenitor T cells were sorted and adoptively transferred into Thy1.1 congenic mice. Two weeks later, the recipient mice were infected *i.p.* with VV-OVA. After various days post-infection, the transferred T cells from the spleen and LNs were analysed. Five mice were used for each time point. (*a*) Differentiation of T cells from HSCs *in vitro*. Morphology of cell differentiation on days 0, 7 and 14. Expression of CD3, TCRβ5 and c-Myc at day 14 was determined by flow cytometry or immunoblot. Data are representative of three independent experiments. In Western blots, β-actin was used as internal control. For Western blots belonging to the same experiment, bands pertaining to different proteins were cropped from the same blot. (*b*) Phenotypic analysis of HSC-derived CD8^+^ T cells. After two weeks of adoptive transfer, CD8^+^ T cells from the pooled LNs and spleens were analysed, gating on Thy1.2^+^ populations. Data are representative of three independent experiments. (*c*) The frequencies of Thy1.2^+^ T cells at day 35 post-infection of VV-OVA, gating on CD8^+^ cells. Data are representative of three independent experiments (*p* < 0.001 between Wt and *Cd28*^−/−^ or *Ox40^−/−^* with Mig; *p* < 0.01 between Wt and *Cd28*^−/−^ or *Ox40^−/−^* with Mig-Myc; two-way ANOVA). (*d*) Functional analysis using IFN-γ. At day 35 post-infection of VV-OVA, splenocytes were stimulated with OVA peptide for intracellular IFN-γ staining, gating on Thy1.2^+^ cells. Data are representative of three independent experiments (*p* < 0.001 between Wt and *Cd28*^−/−^ or *Ox40^−/−^* with Mig; *p* < 0.05 between Wt and *Cd28*^−/−^ or *Ox40^−/−^* with Mig-Myc; two-way ANOVA). (*e*) Numbers of CD8^+^ Thy1.2^+^ T cells at indicated day post-infection of VV-OVA. Data are represented as the mean ± s.e.m. (*p* < 0.01 between Mig and Mig-Myc in *Ox40^−/−^* on days 14, 21 and 28; two-way ANOVA). (*f*) Numbers of CD8^+^ Thy1.2^+^ T cells at day 35 post-infection of VV-OVA. Data are represented as the mean ± s.e.m. from three independent experiments (*p < 0.05; two-way ANOVA).

We first used a system that combined *in vitro* stimulation and *in vivo* development of haematopoietic stem cells (HSCs). HSCs were retrovirally transduced with the c-Myc gene to generate naive CD8^+^ T cells over-expressing c-Myc. CD117^+^ HSCs from the bone marrow of Wt *Cd28*^−/−^ or *Ox40^−/−^* mice were cultured on SNL feeder cells and transduced with retroviral vectors expressing GFP alone, or GFP with c-Myc. GFP^+^ HSCs were sorted and cultured on OP9-DL1/DL4 cells expressing Notch ligands DL1 and DL4 in the presence of IL-7 and Flt3 L. After 14 days of co-culture, CD3^+^ TCRβ5^+^ progenitor T cells were sorted and over-expression of c-Myc was confirmed by immunoblots (figure 4*a*). The sorted CD3^+^ TCRβ5^+^ progenitor T cells were adoptively transferred into Thy1.1 congenic mice. After a two-week maturation *in vivo*, HSC-derived CD8^+^ T cells, gating on Thy1.2^+^ cells, highly expressed CD62 L, CCR7 and CD127, but not CD44, CCR5 and CD122, which are typical phenotypes of naive T cells (figure 4*b*). In addition, the naive T cells highly expressed c-Myc. These results determined the development of naive c-Myc-expressing CD8^+^ T cells from HSCs of Wt *Cd28*^−/−^ or *Ox40^−/−^* mice.

We then investigated whether naive c-Myc-expressing CD8^+^ T cells from *Cd28^−/−^* or *Ox40^−/−^* mice could efficiently differentiate into memory CD8^+^ T cells during primary OVA-VV infection. At day 35 post-infection of VV-OVA, virus-specific memory CD8^+^ T cells from the spleens and LNs of mice were determined, gating on Thy1.2^+^ cells. The reduced number and defective function of virus-specific memory cells from *Cd28*^−/−^ or *Ox40*^−/−^ mice was significantly improved with over-expression of c-Myc (figure 4*c*,*d*). Moreover, the decrease in the frequencies of virus-specific T cells from *Cd28*^−/−^ or *Ox40*^−/−^ mice on days 7 to 35 were substantially but not completely reversed when over-expressing c-Myc (figure 4*e*,*f*).

Collectively, these results demonstrated that the regulation of c-Myc from costimulatory signals affects the expansion (CD28) and the contraction (OX40) of virus-specific memory CD8^+^ T cells during the primary viral infection. The c-Myc-mediated increase in memory cells in *Cd28*^−/−^ is likely due to the increase of cell number in effector cells, and in *Ox40*^−/−^ is due to the inhibition of cell death in effector cells.

### 2.5. C-Myc-mediated regulation of memory CD8^+^ T cells through survivin

C-Myc mediates a number of T-cell functions, including proliferation, homeostasis and metabolic reprogramming. We showed that the sustained survivin expression from costimulatory signals controls T-cell expansion [13]. Therefore, we investigated whether c-Myc-mediated regulation of T-cell memory through survivin.

**Figure 5.**
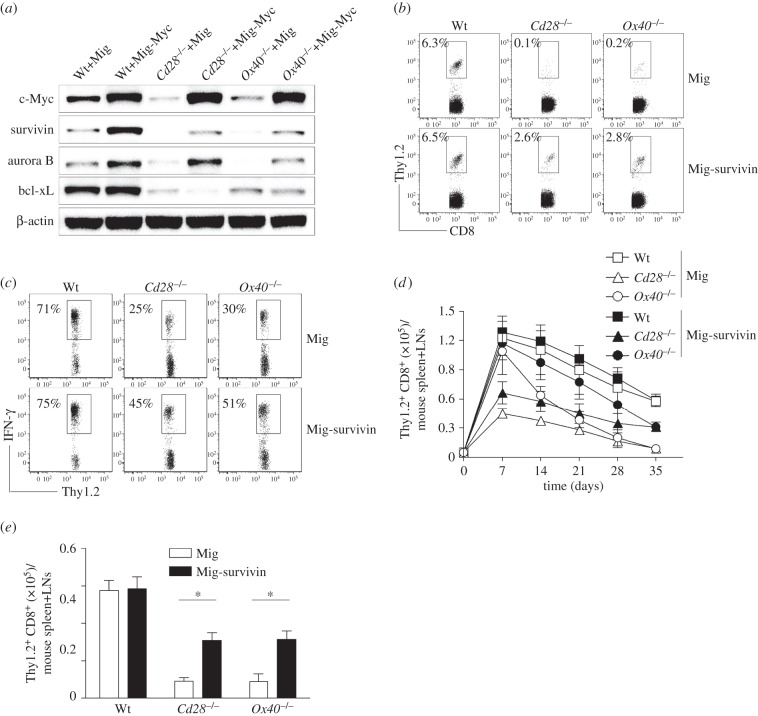
C-Myc regulates the expression of survivin to modulate the development of memory CD8^+^ T cells. Naive CD8^+^ TCRVβ5^+^ T cells from OT-I, OT-I/*Cd28*^−/−^ or OT-I/*Ox40^−/−^* mice were stimulated with peptide and APCs. On day 2/3, T cells were transduced with retroviral vectors expressing GFP alone (Mig) or GFP with survivin (Mig-survivin). On day 5 of primary culture, 5 × 10^4^ GFP^+^ CD8 cells were sorted and adoptively transferred into Thy1.1 congenic mice that were infected *i.p.* with VV-OVA on the following day. After various days, the transferred T cells from the spleens and LNs were analysed. Five mice were used for each time point. (*a*) C-Myc transduction. On day 5 of primary culture, GFP^+^ T cells were sorted and analysed for c-Myc, survivin, aurora B, bcl-xL and β-actin by immunoblot. Similar data were obtained in three experiments. In Western blots, β-actin was used as internal control. For Western blots belonging to the same experiment, bands pertaining to different proteins were cropped either from the same blot or multiple gels were run under similar experimental conditions. (*b*) The frequencies of Thy1.2^+^ T cells at day 35 post-infection of VV-OVA, gating on CD8^+^ cells. Data are representative of three independent experiments (*p* < 0.001 between Wt and *Cd28*^−/−^ or *Ox40^−/−^* with Mig; *p* < 0.01 between Wt and *Cd28*^−/−^ or *Ox40^−/−^* with Mig-survivin; two-way ANOVA). (*c*) Functional analysis using IFN-γ. At day 35 post-infection of VV-OVA, splenocytes were stimulated with OVA peptide for intracellular IFN-γ staining, gating on Thy1.2^+^ cells. Data are representative of three independent experiments (*p* < 0.001 between Wt and *Cd28*^−/−^ or *Ox40^−/−^* with Mig; *p* < 0.05 between Wt and *Cd28*^−/−^ or *Ox40^−/−^* with Mig-survivin; two-way ANOVA). (*d*) Numbers of CD8^+^ Thy1.2^+^ T cells at indicated day post-infection of VV-OVA. Data are represented as the mean ± s.e.m. (*p* < 0.01 between Mig and Mig-survivin in *Ox40^−/−^* on days 14, 21 and 28; two-way ANOVA). (*e*) Numbers of CD8^+^ Thy1.2^+^ T cells at day 35 post-infection of VV-OVA. Data are represented as the mean ± s.e.m. from three independent experiments (*p < 0.05; two-way ANOVA).

To determine if survivin is regulated by c-Myc, c-Myc gene-transduced Wt, *Cd28*^−/−^ or *Ox40^−/−^* CD8^+^ T cells were examined for the expression of survivin. Gene transduction of c-Myc in CD8^+^ T cells upregulated the expression of survivin and aurora B, but not bcl-xL (figure 5*a*). This observation resembles the reported effects in various transformed cells that c-Myc regulated the expression of survivin [23,24].

Next, we determined if an over-expression of survivin in *Cd28*^−/−^ or *Ox40^−/−^* CD8^+^ T cells could reverse their defective generation of memory T cells during viral infection. Similar to the previous approaches, survivin gene-transduced Thy1.2^+^ CD8 cells were adoptively transferred into Thy1.1 congenic mice that were infected *i.p.* with VV-OVA on the following day. At day 35 post-infection of VV-OVA, virus-specific memory CD8^+^ T cells from the spleens and of mice were analysed, gating on Thy1.2^+^ cells. Over-expression of survivin boosted the number, frequencies and function of virus-specific memory cells, which were reduced in *CD28*^−/−^ or *Ox40*^−/−^ mice (figure 5*b*–*e*). These results correspond to the data demonstrating that the regulation of survivin from costimulatory signalling controls T-cell proliferation and clonal expansion [13,17], which may result in generating more memory progenitors and T cells. In addition, the transgenic expression of survivin compensates for OX40-deficiency in driving Th2 development and allergic inflammation [16]. Taken together, these results suggest that the transcriptional regulation of memory CD8^+^ T cells by c-Myc from costimulatory signals is dependent on survivin.

### 2.6. Over-expression of survivin with bcl-xL, a downstream molecule of NF-κB, reverses defective development of memory CD8^+^ T cells in the absence of costimulatory signals

**Figure 6.**
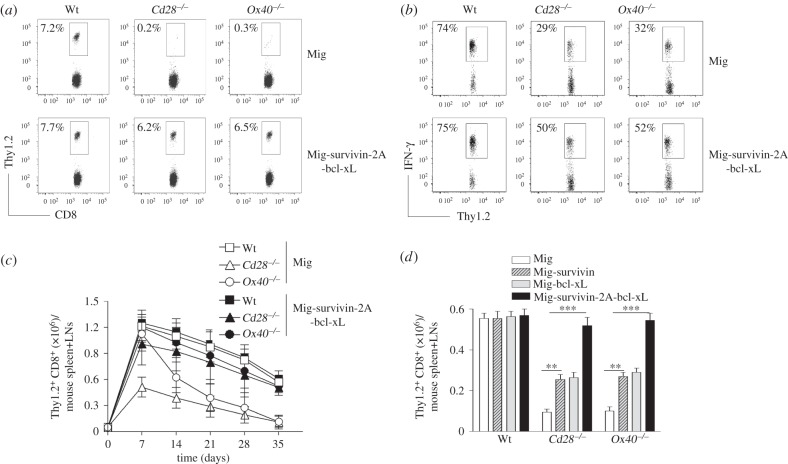
Over-expression of survivin and bcl-xL restored the defect of *Cd28*^−/−^ or *Ox40^−/−^* CD8^+^ T cells in the development of memory. Naive CD8^+^ TCRVβ5^+^ T cells from OT-I, OT-I/*Cd28*^−/−^ or OT-I/*Ox40^−/−^* mice were stimulated with peptide and APCs. On day 2/3, T cells were transduced with retroviral vectors expressing GFP alone (Mig) or GFP with survivin and bcl-xL. On day 5 of primary culture, 5 × 10^4^ GFP^+^ CD8 cells were sorted and adoptively transferred into Thy1.1 congenic mice that were infected *i.p.* with VV-OVA on the following day. After various days, memory progenitors or memory T cells from the spleen and LNs were analysed. Five mice were used for each time point. (*a*) The frequencies of Thy1.2^+^ T cells at day 35 post-infection of VV-OVA, gating on CD8^+^ cells. Data are representative of three independent experiments. (*p* < 0.001 between Wt and *Cd28*^−/−^ or *Ox40^−/−^* with Mig; *p* < 0.01 between Wt and *Cd28*^−/−^ or *Ox40^−/−^* with Mig-survivin-2A-bcl-xL; two-way ANOVA). (*b*) Functional analysis. At day 35 post-infection of VV-OVA, splenocytes were stimulated with OVA peptide for intracellular IFN-γ staining, gating on Thy1.2^+^ cells. Data are representative of three independent experiments (*p* < 0.001 between Wt and *Cd28*^−/−^ or *Ox40^−/−^* with Mig; *p* < 0.05 between Wt and *Cd28*^−/−^ or *Ox40^−/−^* with Mig-survivin-2A-bcl-xL; two-way ANOVA). (*c*) Numbers of CD8^+^ Thy1.2^+^ T cells at indicated day post-infection of VV-OVA. Data are represented as the mean ± s.e.m. (*p* < 0.001 between Mig or Mig-survivin-2A-bcl-xL in *Cd28*^−/−^ or *Ox40^−/−^* on days 14, 21, 28 and 35; two-way ANOVA). (*d*) Numbers of CD8^+^ Thy1.2^+^ T cells at day 35 post-infection of VV-OVA. Data are represented as the mean ± s.e.m. from three independent experiments (**p < 0.01, ***p < 0.001; two-way ANOVA).

Costimulatory signals have been implicated in the proliferation and survival of T cells, which can regulate the generation, maintenance and quantity of virus-specific memory CD8^+^ T cells [18]. The defective proliferation and survival of *Cd28*^−/−^ or *Ox40*^−/−^ CD8^+^ T cells may be due to lack of critical factors which result in reduced generation of memory CD8^+^ T cells during viral infection. Over-expression of survivin, a downstream molecule of c-Myc, or CA-IKKβ, a downstream molecule of NF-κB that compensates for the deficiency of Nfkb1 in canonical NF-κB activity [17], substantially reversed the defective development of memory CD8^+^ T cells in the absence of CD28 or OX40 during viral infection (figures 3–5). To show the essential role of proliferation by c-Myc and survival by Nfkb1 controlled by costimulatory signals for the generation of virus-specific memory CD8^+^ T cells, we then investigated a reversion of proliferation and survival of *Cd28*^−/−^ or *Ox40*^−/−^ CD8^+^ T cells similar to Wt CD8^+^ T cells by over-expression of both survivin and bcl-xL [15]. We previously showed that costimulation targets Nfkb1, which promotes the expression of a downstream molecule bcl-xL to control T-cell survival [15,17]. Thy1.2^+^ CD8^+^ T effector cells expressing both survivin and bcl-xL were adoptively transferred into Thy1.1 congenic mice that were infected *i.p.* with VV-OVA on the following day. Expression of survivin and bcl-xL caused an increase in number, frequencies and function of virus-specific memory cells from *Cd28*^−/−^ or *Ox40*^−/−^ CD8^+^ T cells as compared to *Cd28*^−/−^ or *Ox40*^−/−^ CD8^+^ T cells alone (figure 6). These results show that the defective generation of virus-specific memory CD8^+^ T cells in *Cd28*^−/−^ or *Ox40*^−/−^ mice were largely due to the defective expansion and/or survival during the contraction of the virus-responding cells, in which the reduced expression of c-Myc caused less proliferation in both phases of memory development.

### 2.7. C-Myc modulates the generation of memory CD8^+^ T cells during viral infection

**Figure 7.**
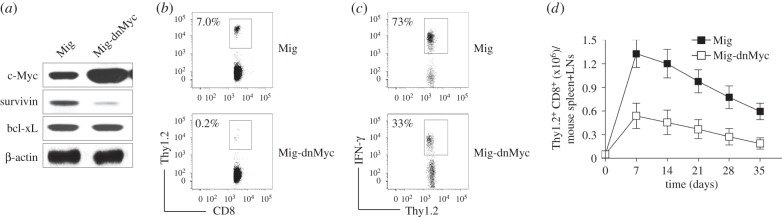
Dominant-negative c-Myc inhibits the generation of memory CD8^+^ T cells. Naive CD8^+^ TCRVβ5^+^ T cells from OT-I TCR Tg mice were stimulated with peptide and APCs. On day 2/3, T cells were transduced with retroviral vectors expressing GFP alone (Mig) or GFP with dominant-negative (dn) c-Myc (Mig-dnMyc). On day 5 of primary culture, 5 × 10^4^ GFP^+^ CD8 cells were sorted and adoptively transferred into Thy1.1 congenic mice that were infected *i.p.* with VV-OVA on the following day. After various days, memory progenitors or memory T cells from the spleen and LNs were analysed. Five mice were used for each time point. (*a*) DnMyc transduction. On day 5 of primary culture, GFP^+^ T cells were sorted and analysed for c-Myc and β-actin by immunoblot. Similar data were obtained in three experiments. In Western blots, β-actin was used as internal control. For Western blots belonging to the same experiment, bands pertaining to different proteins were cropped either from the same blot or multiple gels were run under similar experimental conditions. (*b*) The frequencies of Thy1.2^+^ at day 35 post-infection of VV-OVA, gating on CD8^+^ cells. Data are representative of three independent experiments (*p* < 0.001 between Mig and Mig-dnMyc; one-way ANOVA). (*c*) Functional analysis using IFN-γ. At day 35 post-infection of VV-OVA, splenocytes were stimulated with OVA peptide for intracellular IFN-γ staining, gating on Thy1.2^+^ cells. Data are representative of three independent experiments (*p* < 0.01 between Mig and Mig-dnMyc; one-way ANOVA). (*d*) The absolute numbers of CD8^+^ Thy1.2^+^ at indicated day post-infection of VV-OVA. Data are represented as the mean ± s.e.m. (*p* < 0.001 between Mig and Mig-dnMyc on days 7, 14, 21, 28 and 35; two-way ANOVA).

To directly demonstrate a requirement of c-Myc for the generation of virus-specific memory CD8^+^ T cells, we used a retroviral vector containing a dominant-negative c-Myc (dn-Myc), which contains a deletion of amino acids 106–143 in the transactivation domain. Immunoblots presented the increased levels of c-Myc after the expression of dn-Myc and no effect on the expression of bcl-xL. Of note, immunoblots showed a reduced expression of survivin in CD8^+^ TCRVβ5^+^ T cells from Wt mice (figure 7*a*).

Thy1.2^+^ CD8^+^ T effector cells expressing dn-Myc were adoptively transferred into Thy1.1 congenic mice that were infected *i.p.* with VV-OVA on the following day. Dn-Myc cells had dramatically decreased the numbers (figure 7*b*), function (figure 7*c*) and frequencies of virus-specific memory cells (figure 7*d*) when compared to Wt CD8^+^ T cells, mimicking the defects of *Cd28*^−/−^ or *Ox40*^−/−^ CD8^+^ T cells. Collectively, these data provide strong evidence that c-Myc is required for the generation of efficient virus-specific memory CD8^+^ T cells and that the regulation of c-Myc through costimulatory signalling is critical to the expansion and the contraction of memory CD8^+^ T cells during viral infection.

## 3. Discussion

A number of animal models for viral infections have demonstrated that costimulatory molecules of both the CD28 and the TNFR family members aid in the generation and/or maintenance of virus-specific memory CD8^+^ T cells. In addition to potentially acting in a redundant manner, the membrane-bound costimulatory molecules can provide an opportunity to expand the responses of virus-specific memory CD8^+^ T cells (e.g. CD28) [4,25] and are useful in designing effective vaccines against a variety of chronic viral infections (e.g. OX40) [26,27]. However, the regulating mechanisms of costimulatory signalling in the development of memory T cells remain largely unidentified. In this work, we evidenced that the transcriptional factor c-Myc is a crucial downstream target of the CD28 and the TNFR family members, which regulates the development of virus-specific memory CD8^+^ T cells.

C-Myc is a proto-oncogene that regulates numerous cellular functions, including proliferation, cellular growth, pluripotent stem/progenitor self-renewal and terminal differentiation, immortalization, as well as apoptosis [28–30]. C-Myc is essential during cell and tissue development, because a disruption of the c-Myc gene leads to the early embryonic lethality, and an inactivation of c-Myc in haematopoietic cells leads to the rapid loss of most myeloid and lymphoid lineages [31,32]. Conversely, a transgenic over-expression of c-Myc in different tissues (e.g. liver, breast) causes neoplastic diseases (particularly lymphomas) [33], illustrating the potency of c-Myc as an oncogene. C-Myc is indispensable for the development of T-cell memory; however, existing data suggest that the role of c-Myc in the regulation of virus-specific memory CD8^+^ T cells is in the late/maintenance phase of development, with c-Myc a downstream target of IL-15 [34]. A role for c-Myc in the regulation of memory CD8^+^ T cells at the expansion and the contraction/death phases of differentiation has not been previously determined. Using *Cd28*^−/−^ or *Ox40*^−/−^ mice, we now show that c-Myc is a critical downstream molecule of costimulatory signals, which promotes the responses of memory CD8^+^ T cells.

This conclusion seems not to be in agreement with the data in terms of other transcription factors that are associated with the development of memory CD8^+^ T cells. A genome-wide regulatory network identified numerous transcription factors that may involve in the regulation of memory signature genes. In this study, the top 10 transcription factors involved in the development of memory CD8^+^ T cells included Sox4, Tcf7, Eomes, Bhlhe40, Prdm1, Klf2, Bach2, Runx2, Id2 and Stat4, but not c-Myc [35]. Other studies using deficient animals or patients carrying a dominant-negative mutation, demonstrated that Eomes [7,36], Foxo1 [8,37], Id3 [9], Tcf1 [10] and Stat3 [11] played important roles in the development of memory CD8^+^ T cells. All these different transcriptional factors, which are related to the regulation of memory CD8^+^ T cells, have the ability to prevent the terminal differentiation and/or help maintain the fundamental properties of memory T cells. However, c-Myc is essential for the establishment of a metabolically active and proliferative state in T cells after priming. Other transcription factors may maintain c-Myc-initiated cellular activation to maximize clonal expansion and effector differentiation of T cells during acute responses to pathogen infection [38]. In fact, c-Myc-induced transcription factor AP4 is required for host protection mediated by CD8^+^ T cells [39]. C-Myc is one of the central transcriptional factors that control the proliferation of T cells, which is likely to explain why over-expression of c-Myc can substantially reverse the defective development of virus-specific *Cd28*^−/−^ or *Ox40*^−/−^ memory CD8^+^ T cells. Other transcriptional factors that regulate T-cell survival and plasticity such as Nfkb1 [17] may act in a synergistic fashion and control the development of memory CD8^+^ T cells.

C-Myc functions as part a network of interacting elements. C-Myc can heterodimerize with Max, a ubiquitous bHLH-Zip protein that is an obligate heterodimeric partner for c-Myc in mediating its functions, such as proliferation, differentiation and apoptosis [40]. Max-containing heterodimers associate with larger complexes, depending upon the partner protein, resulting in either recruitment or displacement of the basal transcriptional machinery. Myc–Max heterodimers activate transcription through interactions with transcriptional cofactors (i.e. TRRAP, BAF53) and their associated enzymes such as histone acetyltransferases and/or ATPase/helicase complexes [41,42]. Under physiological conditions, Myc–Max transcriptional regulation controls proliferation and cell cycle [30,43]. However, little is known about downstream targets of c-Myc in relation to T-cell proliferation, especially when driven by costimulatory signalling. We previously identified that survivin plays an essential role in costimulation-driven T-cell expansion/proliferation and is associated with the quantity of memory T cells [13,15]. It is likely that c-Myc through survivin mediates such regulation. There are three studies using human cancer or transformed cells, which reported the transcriptional regulation of survivin by c-Myc [23,24,44]. These observations agree with our result that c-Myc can regulate survivin in primary T cells.

Costimulatory signals from the CD28 and TNF family members affect different phases of the immune response during the generation of memory CD8^+^ T cells. Several members of these families play important roles in the expansion, the contraction/death and the maintenance phases of memory T cells. Our previous studies have shown that during the primary stimulation, CD28 costimulation regulates the expansion while OX40 costimulation modulates the contraction/death phase of memory CD8^+^ T cells by regulating the magnitude of the primary response [3,20,21]. These observations highlight that costimulatory signalling affects the quantity of memory CD8^+^ T cells. In this study, CD28 or OX40 costimulation regulates c-Myc that impacted on the primary expansion or the contraction of memory CD8^+^ T cells, indicating that c-Myc has a distinct function in relation to CD28 and OX40. Our results further suggest that costimulatory signalling is a potential controlling mechanism of the quantity of memory CD8^+^ T cells through the transcriptional regulation of c-Myc. However, because costimulatory signalling also regulates the transcriptional factor Nfkb1, which is associated with a number of T-cell activities such as proliferation, survival and maintenance of homeostasis during the generation of memory CD8^+^ T cells, CD28 and TNF family members are likely to modulate all phases of the development of memory CD8^+^ T cells.

In addition to transcriptional regulation, costimulation-mediated epigenetic modifications, including DNA methylation, histone modifications and reorganizations of chromatin structure, may affect the development of memory CD8^+^ T cells. An early observation suggested that the signals from CD28 induced a stable epigenetic modification of the IL-2 promoter, showing CD28 costimulation induced the stable histone acetylation and the loss of cytosine methylation at the IL-2 promoter/enhancer [45]. This was accompanied by an extensive remodelling of the chromatin in this region to a structure highly accessible to DNA-binding proteins [45]. A similar study showed that CD28 costimulation controlled a chromatin-remodelling process of the IL-5 gene during Th2 cell differentiation [46]. Recent work has demonstrated that CD27 costimulation via epigenetic effects suppresses the function of Th17 effector cells and was associated with pathological consequences [47]. All these epigenetic modifications from costimulatory signalling may regulate the development of memory CD8^+^ T cells. Future studies will shed light on this new and potential regulation of memory CD8 T cells.

For adoptive transfer experiments, 5 × 10^4^ OT-I TCR-transgenic cells are much higher than the physiological precursor frequency. T cells may not properly differentiate into memory cells when abnormally high numbers of transgenic cells are given due to competition effects. However, most experiments in these studies are rescue experiments after *in vitro* activation and retroviral transduction; the transfer of a relatively large number of effector cells helps tracking the memory cells [15,48]. In addition, when physiological numbers of virus-specific cells were given to mice followed by infection, similar results were obtained (figures 4 and 5), indicating that the transferred T cells in these studies normally differentiated into memory when high numbers of transgenic cells were used.

In summary, we provide evidence that the transcriptional regulation of c-Myc, together with Nfkb1 from CD28 or TNF family members, controls the development of memory CD8^+^ T cells during viral infection. C-Myc and Nfkb1 separately control downstream targets survivin and/or bcl-xL, and these downstream targets are likely to cooperatively regulate the expansion and the contraction of virus-specific memory CD8^+^ T cells. Other downstream targets such as aurora B, bcl-2 and bfl-1 as well as the epigenetic modification may also be involved in the regulation of virus-specific memory CD8^+^ T cells via costimulatory signalling and their roles remain to be investigated.

## 4. Material and methods

### 4.1. Mice

OT-I TCR-transgenic mice, expressing a TCR composed of variable (Vβ5 and V*α*2) chains responsive to an OVA peptide 257–264 (SIINFEKL), were bred on a C57BL/6 background. *Cd28*^−/−^ or *Ox40*^−/−^ mice were generated as previously described [49]. OT-I TCR-transgenic mice were backcrossed for greater than five generations onto the *Cd28*^−/−^ or *Ox40*^−/−^ background to OT-I/*Cd28*^−/−^ or OT-I/*Ox40*^−/−^ mice. Thy1.1 congenic mice (B6.PL-*Thy1a*/CyJ) were purchased from the Jackson Laboratory (Bar Harbor, ME, USA).

### 4.2. Peptides and antibodies

OVA 257–264 peptide was purchased from the American Peptide Company (Sunnyvale, CA, USA). Survivin (D-8, sc-17779) and Actin (C2, sc-8432) Abs for Western blot were purchased from the Santa Cruz Biotech. Bcl-xL (no. 2762), IKKβ (no. 8943), Aurora B (no. 3094), and C-Myc (no. 9402) Abs were from the Cell Signaling Technology (Beverly, MA, USA). PE/Cy7, PerCP, PerCP/Cy5.5, or APC-conjugated Thy1.2 (Clone 53-2.1), IFN-γ (Clone XMG1.2), CD44 (Clone IM7) Abs were purchased from Biolegend (San Diego, CA, USA).

### 4.3. T cells and APCs

Naive CD8^+^ T cells were purified from the spleen and LNs and APCs were from the spleens of syngeneic non-transgenic mice by depleting T cells as described [15].

### 4.4. T-cell cultures

Cultures were in 48-well plates containing 1 ml RPMI 1640 (Invitrogen) with 10% fetal calf serum (Omega Scientific, CA, USA) [15]. Naive CD8^+^ T cells were plated at a density of 5 × 10^5^ ml^−1^ with 2 × 10^6^ ml^−1^ APCs in the presence of 1 µM of OVA_257–264_ peptide.

### 4.5. PCR array and RT-PCR

Mouse Transcription Factors RT² Profiler PCR Array (Cat. no. PAMM-075A; Qiagen) was performed with RT² SYBR Green Mastermix (Cat. no. 330522; Qiagen) using an ABI StepOnePlus Real-Time PCR System (Life Technologies). Total RNA was extracted using the RNAeasy Mini Kit (Cat. no. 74104; Qiagen) and reverse transcribed using the RT^2^ First Strand Kit (Cat. no. 330401; Qiagen). The cDNA was mixed with an appropriate RT^2^ SYBR Green Mastermix. The mixtures were aliquoted into the wells of the RT^2^ Profiler PCR Array. PCR was performed and finally the relative expression was determined using data from the real-time cycler and the ΔΔCT method. Housekeeping genes (Gusb, Hprt, Hsp90ab1, Gapdh and Actb) were used for RT-PCR normalization.

### 4.6. NF-κB activity

Canonical NF-κB activity was performed with NFκB p50 ELISA Kit (Colorimetric, Signosis). Nuclear proteins were extracted using the NE-PER Nuclear Protein Extraction Kit (Pierce). The activated NF-κB was detected with a specific antibody against p50 subunit, an HRP-conjugated secondary antibody and determined the optical density with a microplate reader at 450 nm.

### 4.7. Retroviral transduction

MIG-Myc-IRES-GFP (Mig-Myc) was obtained from Addgene, MIG-dn-Myc (Δ106–143)-IRES-GFP (Mig-dnMyc) was generated using the phusion site-directed mutagenesis kit (Thermo Scientific no. F-541). MIDR-Myc-IRES-DsRed (MiDR-Myc) [50], MIG-survivin-IRES-GFP (Mig-survivin) [13], MIG-CA-IKKβ-IRES-GFP (Mig-IKKβ) [17] and MIG-survivin-2A-bcl-xL-IRES-GFP (Mig-survivin-2A-bcl-xL) [15] were generated previously. Retroviral transduction was performed as described after 2–3 days of primary cultures of T cells or HSCs [13,50].

### 4.8. Differentiation of naive T cells from HSCs

CD117^+^ HSCs from the bone marrows of OT-I, OT-I/*Cd28*^−/−^ or OT-I/*Ox40^−/−^* mice were cultured on SNL feeders [50] and transduced with retroviral vectors expressing GFP alone, or GFP with c-Myc. GFP^+^ HSCs were sorted and cultured on OP9-DL1/DL4 cells expressing Notch ligands DL1 and DL4 in the presence IL-7 and Flt3 L. After 14 days, CD3^+^ TCRβ5^+^ progenitor T cells were sorted and adoptively transferred into Thy1.1 congenic mice. Two weeks later, after progenitor T-cell transfer, CD8^+^ T cells from the pooled LNs and spleens were analysed, gating on Thy1.2^+^ populations.

### 4.9. Adoptive transfer

Primed T cells cultured with the specific Ag for 5 days and retrovirally transduced on days 2 and 3, were sorted based on GFP and CD8 expression. In a sex-matched fashion, the sorted cells were injected intravenously into six-week-old naive Thy1.1 congenic mice, which were infected with viruses 1 day later. Numbers of the transferred T cells were calculated based on total cell numbers in the spleen and LNs (popliteal, inguinal, brachial, axillary and superficial cervical), together with the percentages of Thy1.2^+^ CD8^+^ cells visualized by flow cytometry.

### 4.10. Viral infection

VV-OVA infection was performed by an intraperitoneal injection of viruses (2 × 10^6^ PFU mouse^−1^) as described [51].

### 4.11. Functional analysis of memory T cells

At day 35 post-infection, splenocytes were stimulated with 1 µM OVA_257–264_ peptide for 7 h. IFN-γ was analysed by intracellular cytokine staining, gating on Thy1.2^+^ cells.

### 4.12. Immunoblot

Live CD8^+^ cells were recovered by Ficoll treatment and positive selection with anti-CD8 microbeads (Miltenyi Biotec, Inc.). Cell lysates were extracted and used for Western blotting as described [13].

### 4.13. Statistics

One-way or two-way ANOVA was used for statistical analysis between groups and significance was set at 5%. All statistics were calculated using GraphPad Prism (San Diego, CA, USA).
